# Pathological Margin Clearance and Survival After Pancreaticoduodenectomy in a US and European Pancreatic Center

**DOI:** 10.1245/s10434-018-6467-9

**Published:** 2018-04-12

**Authors:** Stijn van Roessel, Gyulnara G. Kasumova, Omidreza Tabatabaie, Sing Chau Ng, L. Bengt van Rijssen, Joanne Verheij, Robert M. Najarian, Thomas M. van Gulik, Marc G. Besselink, Olivier R. Busch, Jennifer F. Tseng

**Affiliations:** 1 Surgical Outcomes Analysis & Research, Beth Israel Deaconess Medical Center, Harvard Medical School, Boston, MA USA; 2 Department of Surgery, Cancer Center Amsterdam, Academic Medical Center Amsterdam, Amsterdam, The Netherlands; 3 Department of Pathology, Cancer Center Amsterdam, Academic Medical Center Amsterdam, Amsterdam, The Netherlands; 4 Department of Pathology, Beth Israel Deaconess Medical Center and Harvard Medical School, Boston, MA USA; 5 Department of Surgery, Boston Medical Center, Boston University School of Medicine, Boston, MA USA

## Abstract

**Background:**

The optimal definition of a margin-negative resection and its exact prognostic significance on survival in resected pancreatic adenocarcinoma remains unknown. This study was designed to assess the relationship between pathological margin clearance, margin type, and survival.

**Methods:**

Patients who underwent pancreaticoduodenectomy with curative intent at two academic institutions, in Amsterdam, the Netherlands, and Boston, Massachusetts, between 2000 and 2014 were retrospectively evaluated. Overall survival, recurrence rates, and progression-free survival (PFS) were assessed by Kaplan–Meier estimates and multivariate Cox proportional hazards analysis, according to pathological margin clearance and type of margin involved.

**Results:**

Of 531 patients identified, the median PFS was 12.9, 15.4, and 24.1 months, and the median overall survival was 17.4, 22.9, and 27.7 months for margin clearances of 0, < 1, and ≥1 mm, respectively (all log-rank *p* < 0.001). On multivariate analysis, patients with a margin clearance of ≥1 mm demonstrated a survival advantage relative to those with 0 mm clearance [hazard ratio (HR) 0.71, *p* < 0.01], whereas survival was comparable for patients with a margin clearance of < 1 mm versus 0 mm (HR: 0.93, *p* = 0.60). Patients with involvement (0 or < 1 mm margin clearance) of the SMV/PV margin demonstrated prolonged median overall survival (25.7 months) relative to those with SMA involvement (17.5 months).

**Conclusions:**

In patients undergoing pancreaticoduodenectomy for pancreatic adenocarcinoma, a margin clearance of ≥1 mm correlates with improved survival relative to < 1 mm clearance and may be a more accurate predictor of a complete margin-negative resection in pancreatic cancer. The type of margin involved also appears to impact survival.

**Electronic supplementary material:**

The online version of this article (10.1245/s10434-018-6467-9) contains supplementary material, which is available to authorized users.

Pancreatic cancer is currently the third-leading cause of cancer-related mortality in the United States with an estimated annual incidence of 16.5 per 100,000 individuals in 2017 and an annual mortality of 13.3 per 100,000.^1^ Surgery combined with adjuvant therapy offers the best chance for long-term survival, but even the minority of patients with localized disease amenable to curative-intent resection face a 5-year survival that rarely exceeds 20–25%.^2^–^4^ Resection margin status is a key prognosticator after surgery and often is used to stratify patients enrolled in clinical trials of adjuvant therapy.^2^,^5^ However, controversy exists as several studies have failed to demonstrate a survival benefit for patients with a margin-negative resection.^6^–^8^ The exact prognostic significance of margin involvement remains fairly understudied in the current literature.

Rates of microscopically positive resection margins (R1) and local recurrence vary widely in the literature, contributing to the unclear relationship between margin status and survival.^6^,^9^–^11^ Traditionally, the proportion of margin-negative resections has been recognized as an indicator of surgical quality; however, some argue that high R1 rates may be considered a reflection of high-quality pathological assessment, rather than inadequate surgical technique.^12^,^13^ Microscopically negative resection margins typically refers to the absence of tumor cells at the inked resection margin (margin clearance > 0 mm) according to College of American Pathologists (CAP), but many European centers define a margin-negative resection as no tumor cells within 1 mm of the resection margin, according to the UK Royal College of Pathologists (RCPath).^14^,^15^ Inconsistency persists in the definitions and protocols used with potentially crucial consequences for the generalizability of outcomes of currently ongoing randomized, controlled trials on resected pancreatic cancer.^16^

The present study was designed to elucidate the relationship between pathological margin clearance and outcomes after pancreaticoduodenectomy (PD) for pancreatic ductal adenocarcinoma in a multicenter cohort from the Netherlands and the United States to establish a clinically meaningful R1 definition which best correlates with survival. Additionally, the prognostic significance of various resection margins on clinical outcome are evaluated.

## Methods

### Data Collection

Patients who underwent PD between 2000 and 2014 at Academic Medical Center, Amsterdam, the Netherlands (AMC) and between 2001 and 2014 at Beth Israel Deaconess Medical Center, Boston, Massachusetts (BID) were retrospectively identified from prospectively maintained institutional databases. Only patients with a histopathologic diagnosis of pancreatic ductal adenocarcinoma (histology ICD-O-3 codes 8140 and 8500) were included. Patients who received neoadjuvant therapy, had metastatic disease, or grossly positive resection margins (R2 resections) were excluded. All pathology reports were reviewed, and additional data on margin clearance and the specific margins involved were extracted. Ambiguities in the pathology report were resolved in consultation with a senior pancreatic pathologist at each institution (J.V. and R.M.N.) and specimens were retrospectively reevaluated, if necessary.

### Pathological Assessment

The resection margins were postoperatively either marked by the surgeon and inked by the pathologist or immediately inked by the surgeon, followed by fixation of the specimen in formalin. Throughout the study period, different grossing techniques for margin assessment were used at both institutions, including the protocols previously described by Adsay and Verbeke.^17^,^18^ The routinely evaluated margins with all grossing protocols used included the pancreatic neck margin, the superior mesenteric artery (SMA)/uncinate margin, the superior mesenteric vein (SMV)/portal vein (PV) margin, the enteric margins, and the bile duct margin. The posterior retroperitoneal/radial margin was routinely assessed at BID, but not until 2009 at AMC. The examination of the anterior margin gradually became part of the routine margin assessment over time at both institutions.

Margin clearance was defined as the distance from the tumor to the nearest resection margin and reported in millimeters (mm). Because the pancreatic neck margin was sectioned and examined parallel to this resection margin (en face), measuring the exact margin clearance was not possible at either institution. Instead, only a determination of involvement or uninvolvement by tumor was recorded for this margin. All other margins were assessed perpendicularly, allowing the pathologist to define margin clearance of 0 mm (tumor cells at inked margin), < 1 mm (tumor cells > 0 mm but < 1 mm from margin) or ≥1 mm (tumor cells ≥ 1 mm from margin). At BID, the margins were assessed without shaving them off the specimen, enabling the pathologist to differentiate margin clearances beyond 1 mm (i.e., 1–2 mm vs. > 2 mm). Tumors were pathologically staged according to the American Joint Committee on Cancer (AJCC) 7th edition.^19^ In the analysis of various positive resection margins, we considered a margin clearance of less than 1 mm as margin-positive (RCPath definition) to ensure consistency within the entire cohort. Patients with a margin clearance of ≥1 mm were considered as margin-negative, including patients with stated negative margins in the pathology report but missing reported margin clearances (*n* = 12).

### Statistical Analysis

SAS software version 9.4 (SAS Institute, Cary, NC) was used for all statistical analyses. Clinical and pathological characteristics were compared using Chi square, or Fisher’s exact test if any cell frequencies were < 5. Numeric data were presented as medians and interquartile ranges (IQR). The primary outcome was overall survival, calculated as the time in months between date of surgery and date of death, or censored at the date of last follow-up. Survival was assessed using the Kaplan–Meier method and log-rank tests. Additional endpoints included progression-free survival, where recurrence was defined as radiographic or pathological evidence of disease progression. The site of recurrence was also collected, including recurrence in the resection bed (local recurrence), in the liver and lung, or a combination (local and distant recurrence). Patients with an isolated positive pancreatic neck margin were excluded (*n* = 27) from the analysis of margin clearance due to the parallel margin assessment.

Multivariate analysis was performed using a Cox proportional hazards model. All patients with one or more missing variables were excluded from multivariate analysis (*n* = 22), as were those with an isolated positive pancreatic neck margin (*n* = 27). A subset analysis of the BID cohort was performed, because further differentiation of margin clearance was possible (i.e., 1–2 mm and > 2 mm) due to the margin assessment technique. Variables that violated the proportional hazard assumption were accounted for by stratification. A two-tailed *p* < 0.05 was considered statistically significant.

This study was approved by the Institutional Review Boards of both institutions. Study data were collected and managed using the REDCap (Research Electronic Data Capture) electronic data capture tools hosted at BID.^20^

## Results

### Characteristics and Outcomes of the Initial Cohort

The final cohort comprised 531 patients, of whom 255 (48.0%) and 276 (52.0%) underwent PD at AMC and BID, respectively. Baseline demographics and clinicopathological characteristics are shown in Table 1. Median age was 66 years [interquartile (IQR) 59–73], and 51.8% of the patients were male. Cohorts of both institutions were comparable in terms of age, sex, and the proportion with positive lymph nodes. In addition to other baseline differences, a higher proportion of the patients treated at BID received adjuvant therapy (70.6% vs. 54.9%), and they were more likely to receive radiotherapy in addition to chemotherapy compared with those treated at AMC (49.6% vs. 4.3%). The 90-day mortality was 2.8% and 1.1% (*p* = 0.21), and the median overall survival was 23.6 and 23.5 months (*p* = 0.34) for patients treated at AMC and BID, respectively. The median follow-up time was 49.9 months (IQR 32.3–81.5) for survivors.

**Table 1:** Baseline characteristics of the initial cohort by institution

	AMC (*n* = 255)	BID (*n* = 276)	*p* value
Age (year)			
< 65	115 (45.1%)	117 (42.4%)	0.53
≥ 65	140 (54.9%)	159 (57.6%)	
Sex			
Male	135 (52.9%)	140 (50.7%)	0.61
Female	120 (47.1%)	136 (49.3%)	
ASA score			
ASA I	50 (19.6%)	0 (0%)	< 0.0001
ASA II	150 (58.8%)	92 (33.3%)	
ASA III	39 (15.3%)	175 (63.4%)	
ASA IV	16 (6.3%)	9 (3.3%)	
Type surgery			
PPPD	218 (85.5%)	28 (10.1%)	< 0.0001
Whipple	37 (14.5%)	248 (89.9%)	
Vascular resection			
Yes	53 (20.8%)	24 (8.7%)	< 0.0001
No	202 (79.2%)	252 (91.3%)	
Adjuvant therapy			
Chemoradiation	11 (4.3%)	137 (49.6%)	< 0.0001
Only chemotherapy	129 (50.6%)	52 (18.8%)	
Only radiotherapy	0 (0%)	6 (2.2%)	
No adjuvant therapy	112 (43.9%)	81 (29.3%)	
Unknown	3 (1.2%)	0 (0%)	
AJCC T stage			
T1	17 (6.7%)	22 (8.0%)	0.02
T2	54 (21.2%)	34 (12.3%)	
T3/T4	184 (72.2%)	220 (79.7%)	
AJCC N stage			
N0	54 (21.2%)	77 (27.9%)	0.07
N1	201 (78.8%)	199 (72.1%)	
Tumor differentiation			
Well	16 (6.3%)	58 (21.0%)	< 0.0001
Moderate	160 (62.8%)	160 (58.0%)	
Poorly/undifferentiated	73 (28.6%)	56 (20.3%)	
Unknown	6 (2.4%)	2 (0.7%)	
Margin clearance^a^			
0 mm	63 (26.0%)	67 (25.6%)	< 0.0001
< 1 mm	64 (26.5%)	38 (14.5%)	
≥ 1 mm	115 (47.5%)	145 (55.3%)	
Not reported	0 (0%)	12 (4.6%)	

*PPPD* pylorus-preserving pancreaticoduodenectomy; *AJCC* American Joint Committee on Cancer

^a^Patients with an isolated positive pancreatic neck margin not included

### Survival Outcomes by Margin Clearance

**Fig. 1:**
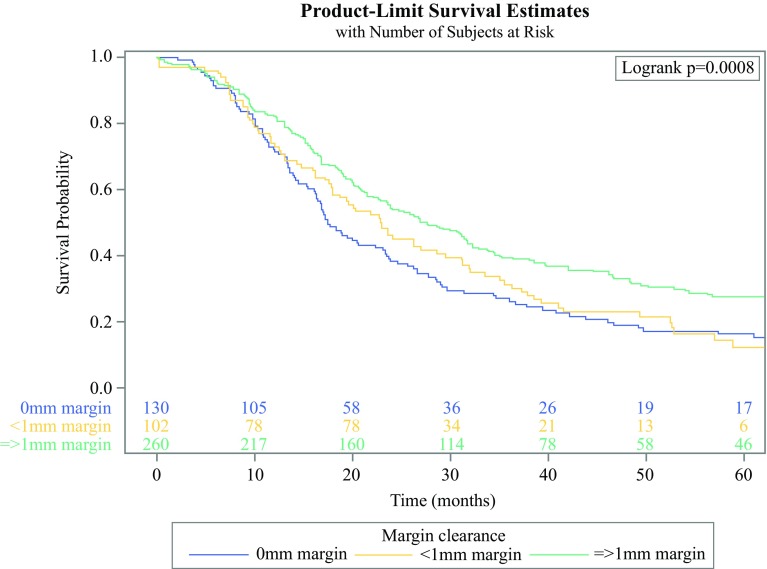
Unadjusted overall survival stratified by margin clearance. Patients with an isolated positive pancreatic neck margin not included in analysis

For patients with a margin clearance of 0 mm, < 1 mm, and ≥1 mm, the median overall survival was 17.4, 22.9, and 27.7 months (*p* < 0.001), and the 5-year survival rate was 16.3, 12.4, and 27.6%, respectively (Fig. 1). Survival was improved in patients with a margin clearance of ≥1 mm relative to those with a clearance of 0 mm (*p* < 0.001) and < 1 mm (*p* = 0.02), whereas there was no significant difference in survival between patients who had a margin clearance of 0 mm versus < 1 mm (*p* = 0.60) on unadjusted analysis. Similarly, recurrence data demonstrated a median PFS of 12.9 and 15.4 months for 0 and < 1 mm margin clearance (*p* = 0.48), whereas patients with a margin clearance of ≥1 mm showed a prolonged median PFS (24.1 months) compared with 0 mm (*p* = 0.001) and < 1 mm (*p* < 0.001). PFS by margin clearance and patterns of recurrence are depicted in the supplementary material (Figs. S1 and S2).

**Fig. 2:**
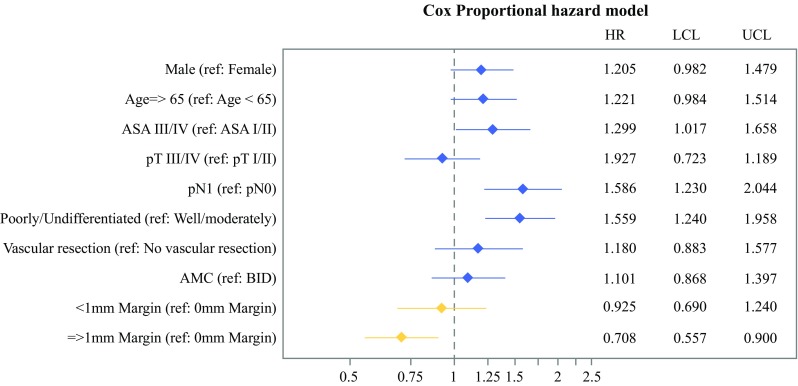
Multivariate Cox proportional hazards model assessing hazard of death for the entire cohort. Model also stratified by type of adjuvant therapy

**Fig. 3:**
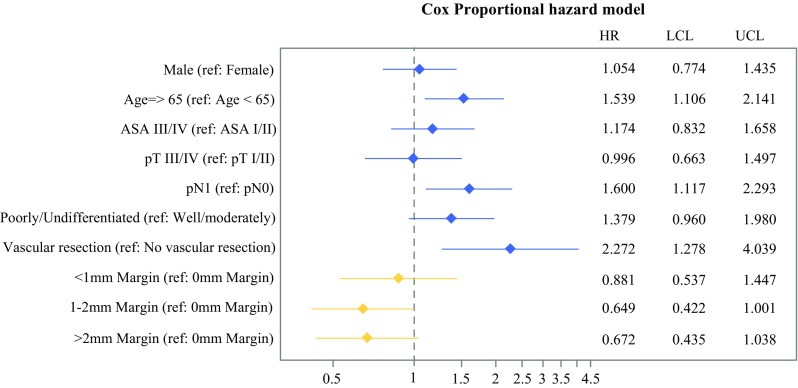
Multivariate Cox proportional hazards model assessing hazard of death for the subset of the BID cohort. Model also stratified by type of adjuvant therapy

On multivariate analysis a higher ASA score, positive lymph nodes, and poorly/undifferentiated tumors were associated with a significantly increased hazard ratio (Fig. 2). Patients with a margin clearance of ≥1 mm demonstrated a survival advantage versus 0 mm (HR 0.71, *p* < 0.01), whereas patients with a margin clearance of  <  1 mm did not demonstrate a survival benefit versus 0 mm (HR 0.93, *p* = 0.60). In the subset analysis of patients treated at BID, there was a trend towards a decreased hazard ratio for patients with both a margin clearance of 1–2 mm (HR 0.65, p = 0.05), as well as > 2 mm (HR 0.67, p = 0.07) compared with the 0 mm clearance group (Fig. 3).

### Survival Outcomes by Positive Margins

Using the definition of < 1 mm to define a positive margin (RCPath definition), 257 patients (48.4%) of the entire cohort had defined margin involvement. The most commonly involved margins were the SMA margin (*n* = 113, 43.6% of all patients with a margin clearance of < 1 mm), the SMV/PV margin (*n* = 77, 29.7%) and the posterior retroperitoneal margin (*n* = 75, 29.0%), which also were associated with the highest 1-year local recurrence rate (32.0% for the SMA margin, 32.2% for the SMV/PV margin, and 38.4% for the posterior retroperitoneal margin) as shown in Table 2. Patients with a positive SMV/PV margin had a significantly prolonged survival, particularly the subgroup not requiring venous resection (26.3 months, *p* = 0.03), compared with patients with one or more other positive margins (< 1 mm), whereas patients with a positive SMA margin had a trend towards worse survival (17.5 months).

**Table 2:** Overall survival stratified by positive margin status

Specific resection margin	No. of patients	Median survival (mo)	1-year recurrence rate (%)	1-year local recurrence rate (%)	Survival compared with R0° (log-rank *p*)	Survival compared with other R1^a^ (log-rank *p*)
Negative margins (≥ 1 mm clearance)	272	27.2	23.0	10.5	–	–
Positive margin (< 1 mm clearance)						
Any positive margin	259	20.1	41.6	27.5	< 0.001	–
Pancreatic neck margin	55	19.5	43.3	28.7	0.06	0.66
SMA/uncinated margin	113	17.5	46.0	32.0	< 0.0001	0.10
SMV/PV margin (with or w/o VR)	77	25.7	43.1	32.2	0.30	0.04
SMV/PV margin (without VR)	49	26.3	33.9	24.2	0.79	0.03
Posterior retroperitoneal margin	75	16.7	52.4	38.4	< 0.01	0.66
Anterior margin	24	20.1	27.9	16.1	0.26	0.75
Proximal gastric/jejunal margin	8	13.6^b^	65.0^b^	12.5^b^	0.02^b^	0.23^b^
Bile duct margin	5	17.2^b^	60.0^b^	60.0^b^	0.65^b^	0.76^b^

*R0* survival compared to all patients with a margin clearance of ≥1 mm, *VR* venous resection

^a^Other R1: survival compared to patients with one or more other involved margins (< 1 mm)

^b^Estimates may not be reliable due to small numbers

## Discussion

Assessment of pathological margin clearance in this multicenter cohort demonstrated that patients with a margin clearance of ≥1 mm have a survival advantage relative to those with a 0 mm clearance (HR 0.71, *p* < 0.01), while survival was comparable for patients with a margin clearance of < 1 mm versus 0 mm (HR 0.93, *p* = 0.60). Moreover, a similar pattern was found in assessment of PFS by margin clearance. This finding challenges the traditional R0/1 definition of margin clearance after PD. In addition, a positive SMV/PV margin demonstrated a less negative clinical impact on overall survival than involvement of the SMA margin.

Studies investigating the relationship between margin clearance and clinical outcome after resected pancreatic adenocarcinoma have been conflicting. Some studies found a survival advantage for patients with a margin clearance above 1 mm and 1.5 mm.^21^,^22^ Although a recent, single-center study from Germany, evaluating 561 patients, demonstrated a significant survival benefit for patients with a margin clearance of ≤1 mm versus 0 mm on unadjusted (median survival 27.5 vs. 23.4 months, respectively; *p* = 0.01) and multivariate analysis (HR 0.69; 95% CI 0.51–0.94).^23^ However, a detailed description of the pathological margin assessment was not provided.

The clinical impact of different involved margins has also been studied and found significantly decreased survival in patients with involvement of the SMA or SMV/PV margins compared to margin-negative resections.^24^–^26^ However, not all studies evaluated the margins separately, grouping the different margins together either as the medial margin (SMV/PV and SMA margins) or as transection margins (SMV/PV, SMA, and pancreatic neck margins).^24^,^26^ The unresolved matter of margin clearance in the literature may partly be caused by the varying rates of involvement of each margin, presumably as a result of heterogeneity in patient selection, surgical technique, and pathological margin assessment.

This paper provides the first multicenter study of margin clearance in resected pancreatic cancer and addresses the pathological challenges of margin assessment after PD in detail to reach valid conclusions. Follow-up in this study was relatively long (median follow-up of 49.9 months for living patients), leading to more accurate 5-year survival rates. These often are considered as a better reflection of local recurrence than median survival, due to many patients with short survival harboring occult metastases.^27^ Both centers are academic, high-volume pancreatic centers and as previously shown differences in adjuvant therapy regimens did not affect survival outcomes.^28^,^29^

Survival also was found to be related to the type of margin involved with better survival for patients with SMV/PV margin involvement. Clinically, residual disease would indeed be more likely to be expected after a positive SMA margin where extrapancreatic soft tissue adjacent to the SMA is divided. Furthermore, a positive SMV/PV margin may not necessarily imply that tumor cells are left behind. If the pancreas was separated intraoperatively from the SMV/PV without requirement of a venous resection, a positive margin could “merely” involve tumor cells close to the pancreatic serosa at the SMV/PV margin. It remains to be assessed whether margin involvement serves as a marker for local recurrence, poor tumor biology, or both. Within the scope of the current study, margin clearance is a significant prognosticator of recurrence and overall survival.

There are several limitations, mostly inherent to the retrospective nature of this study. There may be potential residual confounding, by not adjusting for CA 19-9 levels, tumor location, and the number of positive lymph nodes. Additionally, there were changes in both pathologic assessment and surgical approach over time. However, these changes were taken into account to the best of our ability with contributions of expert pancreatic pathologists and surgeons to allow for appropriate comparisons. For the analysis of margin clearance, the different resection margins were grouped as one; however, the various resection margins may differently affect outcomes, as evidenced by previous studies.^11^,^24^,^26^ Furthermore, the distinction between macroscopically negative (R0/R1) and positive (R2) margins relies on communication between the surgeon and pathologist. While patients with an R2 resection were excluded from analysis, there may be those with documented R1 disease who had R2 disease, which could have resulted in an underestimation of the benefit of R1 resection. Furthermore, certain anatomical boundaries are considered a surgical limit, for example the SMA margin, which in the case of tumor infiltration results in an inevitable macroscopically positive margin.

This work contributes substantially to the current literature with novel, comprehensible and international data, to achieve consensus on pathological protocols and definitions. Our subset analysis demonstrates a survival benefit for patients with a margin clearance of 1–2 mm compared with 0 mm, which further supports the ≥ 1 mm cutoff for a margin-negative resection. Lastly, detailed descriptions of margin assessments were provided for each institution, something that has mostly been limited or omitted in clinical studies reported to date.

Margin clearance remains an important variable under control of the surgeon. Theoretically, margin clearance is dependent on the extent of the tumor, extent of the surgery, and the proximity of an absolute anatomic boundary, by which the surgeon is eventually limited (i.e., either the SMA or the circumferential surfaces of the pancreas). Our findings show that patients with a close surgical margin clearance of < 1 mm represent a group with similar survival as those with a margin clearance of 0 mm, whereas patients with a margin clearance of ≥ 1 mm demonstrated improved survival.

These results support the ≥1 mm definition (RCPath) for R0 resections in pancreatic cancer, which should be considered for future stratification in randomized, controlled trials. In addition, margin assessment should be standardized by examining all transection and circumferential margins, preferably perpendicularly with extensive sampling to achieve realistic R1 rates, which can be more easily reconciled with the high rates of local recurrence.^18^ Finally, in future prospective studies, data collection on the margin clearance and the specific positive resection margin should become standard practice to evaluate the effect of locoregional adjuvant therapies according to margin clearance and the specific positive resection margins. Furthermore, as the use of neoadjuvant therapy becomes more widely adopted, particularly for borderline resectable disease, the impact of positive resection margins after neoadjuvant therapy has yet to be evaluated.^30^

## Electronic supplementary material

Below is the link to the electronic supplementary material.

Supplementary material 1 (DOCX 63 kb)
