# Supplementary material for: Pathological Margin Clearance and Survival After Pancreaticoduodenectomy in a US and European Pancreatic Center
Source: Ann Surg Oncol. 2018 Apr 12;25(6):1760–7. doi: 10.1245/s10434-018-6467-9 (PMC5928169; doi:10.1245/s10434-018-6467-9)
Supplement: Supplementary file 1 — Supplementary material 1 (DOCX 63 kb) [file 10434_2018_6467_MOESM1_ESM.docx]

**SUPPLEMENTARY MATERIALS**

**FIGURE LEGENDS**

**Figure S1.**

Title: Unadjusted progression-free survival (PFS) stratified by margin clearance.

Caption: * Patients with an isolated positive pancreatic neck margin not included in analysis

**Figure S2.**

Title: Unadjusted progression-free survival (PFS) stratified by site of recurrence for all patients with radiological or pathological evidence of recurrence during follow-up.

**
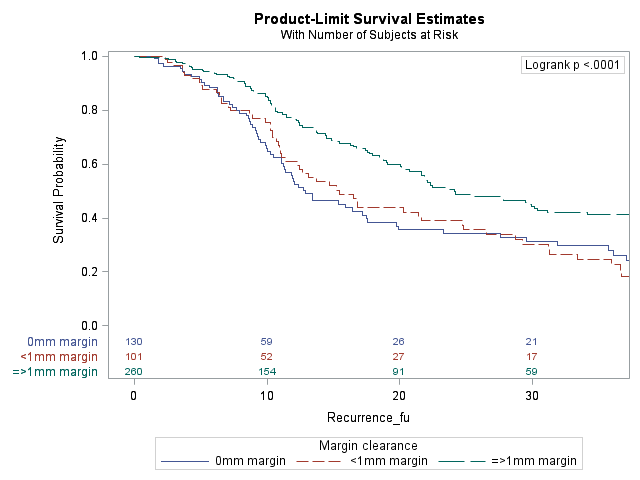
Figure S1.**


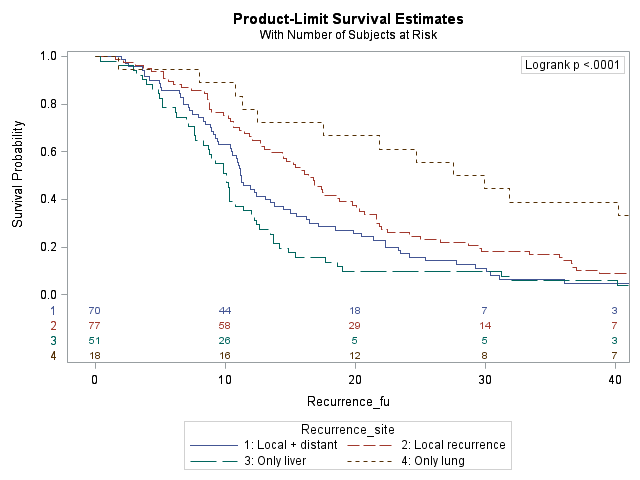
**Figure S2.**
